# Frequency of appearance of anemias at rheumatoid arthritis - a disease of autoimmune genesis (on the data of retrospective study)

**DOI:** 10.1186/ar3608

**Published:** 2012-02-09

**Authors:** Abdumalik N Aripov, Marif SH Karimov, Aida A Eshmurzaeva

**Affiliations:** 1 Tashkent Institute of Postgraduate Medical Education, Tashkent, Uzbekistan; 2 Tashkent Medical Academy, Tashkent, Uzbekistan

## 

The purpose of research is study of offenses of appearance of anemia among rheumatoid arthritis (RA) patients, revealing of their etiologic reasons, as well as the estimation of character of used anti anemia means of medicine on the basis of retrospective analysis of history of disease.

Coming out of above stated histories of illness of RA patients were analyzed to presence of established as accompanying disease of anemia. Results of this analysis are represented on picture as it seen on the presented data, 33,3% of patients with RA anemia is verified as accompanying pathology. Therefore at 1/3 patients with P anemia takes place. The study of etiologic causes of anemia at these patients shows that in 76,6% cases anemia bears ferrous deficit character, 20%- anemia of chronic diseases and only in 3,4% cases - auto immune anemia. Therefore, the majority of patients of RA anemia bears ferrous deficit character.

The high frequency of appearance of ferrous deficit anemia among RA patients, probably is explained by that in conditions of this disease changes of pH happen among gastro duodenal area. Besides, wide use of non steroidal anti inflammatory medicine (NAIM) at RA also may effect to pH of stomach. And in cases of destroyed reaction of ambience change of ferrous assimilation. That fact of ferrous deficit anemia may has independent character at analyzed RA patients is excluded. But on their history of illness it is impossible to determine this fact.

Study of offenses of appearance of anemia at RA patients depending on age categories is evidencing on that 83,4% of patients with anemia comes to patients from 31 to 60 years old, and among patients of 31 to 40 years old appears 25% patients, from 41 to 50 years old - 26,7% and from 51 to 60 years old - 31,7%, accordingly.

Results of these analysis showed that if at patients with debut RA anemia appears at 1,5% cases, than among RA patients with prolongation of anamnesis from 1 to 5 years old, from 5 to 10 years old appears in 33,3%, 28,7% and in 34,8% cases accordingly. Therefore as far as increasing of prolongation of current of RA, specific gravity of patients with anemia increases.

